# Growth and digestive enzyme activities of rohu labeo rohita fed diets containing macrophytes and almond oil-cake

**DOI:** 10.1016/j.anifeedsci.2020.114456

**Published:** 2020-05

**Authors:** R.K. Goswami, A.K. Shrivastav, J.G. Sharma, D.R. Tocher, R. Chakrabarti

**Affiliations:** a Aqua Research Lab, Department of Zoology, University of Delhi, Delhi 110 007, India; b Department of Biotechnology, Delhi Technological University, Bawana Road, Delhi 110042, India; c Institute of Aquaculture, Faculty of Natural Sciences, University of Stirling, Stirling FK9 4LA, Scotland, United Kingdom

**Keywords:** ANOVA, Analysis of Variance, AOAC, Association of Official Analytic Chemists, APHA, American Public Health Association, BBSRC, Biotechnology and Biological Science Research Council, DBT, Department of Biotechnology, DF, Dry fish, DH, Degree of hydrolysis, F, Fishmeal, FAO, Food and Agriculture Organization, FBW, Final body weight, FCR, Feed conversion ratio, FI, Feed Intake, FLM, Fishmeal with *Lemna minor*, FSM, Fishmeal with *Salvinia molesta*, FTC, Fishmeal with *Terminalia catappa*, FTCLMSM, Fishmeal with *Terminalia catappa* Lemna minor, Salvinia molesta, IAEC, Institutional Animal Ethics Committee, IBW, Initial body weight, LM, *Lemna minor*, SGR, Specific growth rate, SM, *Salvinia molesta*, TC, *Terminalia catappa*, TCLMSM, *Terminalia catappa* Lemna minor, Salvinia molesta, WG, Weight gain, Amylase, Chymotrypsin, Duckweed, Growth, *Labeo rohita*, Trypsin

## Abstract

The impact of plant-based diets on the digestive physiology of rohu *Labeo rohita* fingerlings (10.66 ± 0.53 g) was evaluated. A diet with all protein supplied by fishmeal was included as a control (F). Four test diets containing 300 g/kg protein were formulated using the following plant ingredients and fishmeal in a 1:1 blend: almond oil-cake *Terminalia catappa* (FTC), duckweed *Lemna minor* (FLM), water fern *Salvania molesta* (FSM) and combination of these three ingredients (FTCLMSM). The final body weight and specific growth rate were significantly higher in rohu fed diet FLM compared to the other treatments. Significantly lower feed conversion ratio in rohu fed diet FLM showed that diet was utilized efficiently in this feeding regime compared to the other diets. The composition of diets also influenced the digestive enzyme activities of the fish. Thus, amylase, trypsin and chymotrypsin activities were significantly higher in rohu fed diet FLM compared to the rohu fed the other diets. Protease activity was significantly higher in rohu fed diets FTC and F and lipase activity was significantly higher in rohu fed diet FTC compared to the rohu fed the other diets. The inclusion of raw duckweed in feed replaced 300 g/kg of dietary fishmeal without affecting growth.

## Highlights

• Rohu were fed with almond oil-cake/ duckweed/ water fern and fishmeal blend diets.
• Highest growth was found in duckweed-based diet fed rohu.
• Duckweed is a rich source of amino acids for rohu.
• Feed composition influenced amylase, protease and lipase activities.
• Duckweed helped to replace 300 g/kg dietary fishmeal without affecting growth.

## 1 Introduction

Sustainable aquaculture depends on the supply of quality feed to the farmed species. Protein plays significant role in fish nutrition and fishmeal has been traditionally used as a major protein source for the formulation of feed. The availability of quality fishmeal and its cost are two major constrains. Freshwater macrophytes are considered as potentially good sources of protein for formulation of feed for some fish species (Hasan and Chakrabarti, 2009; Chakrabarti, 2017). Several studies show the advantages of use of macrophytes as fish feed ingredients *viz*., feeding of Nile tilapia *Oreochromis niloticus* with fresh *Lemna perpusilla* (Hasan and Edwards, 1992) or diets based on *Azolla africana*, *Spirodela polyrrhiza* (Fasakin et al., 2001) and *A. filiculoides* (Abou et al., 2011; Abou et al., 2013), common carp *Cyprinus carpio* with a diet based on L. *minor* (Yılmaz et al., 2004) and rohu *Labeo rohita* with raw/fermented L. *polyrhiza* (Bairagi et al., 2002) or *Azolla microphylla* and *A. pinnata* (Datta, 2011) or *Ipomoea aquatica* supplemented diets (Ali and Kaviraj, 2018).

*Labeo rohita* rohu (family: Cyprinidae) is an economically important carp that is used extensively in composite fish culture. Rohu is an omnivore, column feeder fish and used in composite fish culture. Digestive tract analysis shows the presence of plant materials (Jhingran, 1991). The present study aims to evaluate the impact of diets supplemented with *Lemna minor* (LM), water fern *Salvania molesta* (SM) and oil-cake of almond *Terminalia catappa* (TC) on the growth and digestive enzyme activities of rohu.

## 2 Materials and methods

### 2.1 Ingredients and their composition

Locally available ingredients were used for the formulation of fish feed. The dry fish (DF), Bombay duck *Harpadon nehereus* was purchased from local fish market Ghazipur, New Delhi, India. The almond oil-cake *T. catappa* (TC) is a low-cost agricultural by-product. It was collected after the extraction of oil from a local oil extraction mill. The duckweed L. *minor* (LM) and water fern *S. molesta* (SM) were cultured in the outdoor cemented tanks using organic manures (Chakrabarti et al., 2018). Macrophytes were harvested, cleaned, air dried and kept in an oven at 40 °C. After drying, fishmeal, almond oil-cake and macrophytes were ground and sieved; fine powder were kept in air tight containers at 4 °C for further use.

Chemical composition of feed ingredients (Table 1 and Table 2) and diets (Table 3) were analyzed following the standard methods. Dry matter content was measured following the method 930.15 of the Association of Official Analytical Chemists (AOAC) (2000). The crude protein content was determined using the method 990.03 (Association of Official Analytical Chemists (AOAC), 2000) with an automated micro-Kjeldhal system (Pelican Instruments, Chennai, India). The nitrogen content was multiplied with 6.25 to calculate the amount of crude protein. The crude lipid content was measured (Folch et al., 1957) gravimetrically after extraction with chloroform/methanol (2:1, v/v). Carbohydrate content was then determined by the subtraction method. Ash contents of samples were determined following the method 942.05 of the Association of Official Analytical Chemists (AOAC) (2000). Energy value was determined following the standard method (Merrill and Watt, 1973).

**Table 1:** Chemical composition of feed ingredients used for the formulation of diets (g/kg as fed). Values are given as Means ± SE (n = 3).

Parameters	F^1^	TC^1^	LM^1^	SM^1^	TCLMSM^1^
Dry matter^2^	948.4 ± 0.78	942.2 ± 0.30	920.1 ± 1.80	928.8 ± 2.46	931.0 ± 0.49
Crude protein	689.0 ± 5.22	457.3 ± 3.03	364.7 ± 2.81	283.6 ± 3.61	351.2 ± 5.32
Crude lipid	82.0 ± 5.20	93.2 ± 2.32	73.9 ± 0.83	48.5 ± 1.20	69.8 ± 1.53
Total carbohydrate	15.4 ± 6.02	325.9 ± 0.10	263.9 ± 6.40	439.7 ± 0.91	362.8 ± 2.62
Crude ash	165.2 ± 3.34	65.8 ± 0.11	217.2 ± 0.24	157.0 ± 0.90	147.2 ± 1.14
Energy value (kcal/kg)^7^	3556.0 ± 32.0	3971.6 ± 23.4	3427.5 ± 24.31	3329.7 ± 28.88	3484.2 ± 25.43

^1^F, Fishmeal; TC, *T. catappa*; LM, *L. minor*; SM, *S. molesta*; TCLMSM, *T. catappa* + *L. minor* + *S. molesta*.

^2^Dry matter = Weight in g (1000 – Moisture) in 1 kg of feed.

^3^Energy (kcal/kg) = [(Crude protein g/kg × 4) + (Crude lipid g/kg × 9) + (Total Carbohydrate g/kg × 4)].

**Table 2:** Amino acid composition of ingredients use in experimental diets as a protein source in (g/kg as fed). Values are given as Means ±SE (n = 3).

Amino acids	F^1^	TC^1^	LM^1^	SM^1^	TCLMSM^1^
Essential					
Arginine (Arg)	45.49 ± 0.021	56.58 ± 0.085	30.60 ± 0.452	17.53 ± 0.021	27.80 ± 0.202
Histidine (His)	18.63 ± 5.747	10.75 ± 0.653	8.94 ± 0.115	6.82 ± 1.727	7.43 ± 0.013
Isoleucine (Ile)	31.81 ± 2.067	18.84 ± 1.953	20.43 ± 0.646	11.90 ± 0.918	15.78 ± 0.426
Leucine (Leu)	55.83 ± 2.543	34.11 ± 2.399	41.32 ± 0.463	21.47 ± 2.065	28.17 ± 0.948
Lysine (Lys)	61.15 ± 1.265	13.51 ± 0.952	26.83 ± 1.614	15.64 ± 0.036	18.41 ± 1.389
Methionine (Met)	20.31 ± 0.016	3.63 ± 0.983	8.59 ± 0.142	5.86 ± 0.042	5.59 ± 0.474
Phenylalanine (Phe)	29.69 ± 0.288	26.54 ± 2.219	25.71 ± 0.344	14.38 ± 0.626	20.24 ± 0.844
Threonine (Thr)	33.41 ± 0.187	16.36 ± 0.492	19.24 ± 1.389	14.37 ± 0.001	15.02 ± 0.393
Tryptophan (Trp)	14.28 ± 0.001	3.86 ± 0.081	3.65 ± 0.107	5.54 ± 0.005	7.00 ± 0.024
Valine (Val)	37.07 ± 2.127	22.36 ± 2.060	26.64 ± 0.966	16.39 ± 1.241	20.41 ± 0.537
Non-essential					
Alanine (Ala)	46.54 ± 1.541	22.60 ± 1.152	28.82 ± 0.410	16.62 ± 1.436	19.72 ± 0.624
Aspartate (Asp)	68.83 ± 2.329	59.61 ± 0.103	37.14 ± 3.722	30.17 ± 0.104	39.98 ± 0.501
Cysteine (Cys)	6.40 ± 0.810	6.94 ± 0.683	3.81 ± 0.321	3.29 ± 0.200	4.22 ± 0.271
Glutamic acid (Glu)	129.48 ± 5.072	147.92 ± 14.353	64.27 ± 1.025	39.01 ± 0.436	78.70 ± 1.488
Glycine (Gly)	43.32 ± 0.610	31.08 ± 1.407	28.61 ± 0.312	15.30 ± 1.105	21.30 ± 0.620
Proline (Pro)	27.41 ± 1.220	20.11 ± 1.147	12.48 ± 0.353	11.59 ± 1.191	15.26 ± 0.611
Serine (Ser)	26.12 ± 0.295	20.61 ± 0.255	23.48 ± 3.209	13.90 ± 1.044	15.33 ± 0.317
Tyrosine (Tyr)	26.06 ± 2.342	16.18 ± 0.832	19.05 ± 1.250	11.36 ± 1.304	13.29 ± 2.411

Free amino acids					
Phosphoserine (p- Ser)	2.26 ± 0.064	3.11 ± 1.086	5.78 ± 0.001	2.19 ± 0.002	1.91 ± 0.337
Taurine (Tau)	2.44 ± 0.173	0.16 ± 0.011	0.41 ± 0.151	0.19 ± 0.031	0.15 ± 0.011
Phospho ethanol amine (PEA)	–	0.24 ± 0.014	0.23 ± 0.066	0.55 ± 0.141	0.41 ± 0.049
Sarcosine (Sar)	5.17 ± 1.399	–	0.97 ± 0.043	–	0.24 ± 0.012
α Amino-n-adipic acid (α - AAA)	2.73 ± 0.101	–	0.45 ± 0.136	0.28 ± 0.001	1.05 ± 0.735
α Amino-n- butaric acid (α - ABA)	–	–	1.50 ± 0.123	–	–
Cystathionine (Cysthi)	3.65 ± 0.107	2.39 ± 0.606	0.93 ± 0.198	1.75 ± 0.069	1.89 ± 0.197
β Alanine (β-Ala)	–	3.51 ± 1.010	1.11 ± 0.204	3.02 ± 0.540	1.00 ± 0.121
β Amino isobutyric acid (beta-AiBA)	17.49 ± 1.001	–	9.71 ± 2.711	3.90 ± 1.415	1.260 ± 0.230
ϒ Amino butyric acid (ϒ - ABA)	3.00 ± 1.016	2.67 ± 1.143	4.05 ± 0.149	2.80 ± 0.151	3.86 ± 0.102
Ethanol amine (EOHNH2)	2.86 ± 0.552	1.65 ± 0.019	1.46 ± 0.043	1.73 ± 0.452	1.60 ± 0.931
Hydroxylysine (Hylys)	4.48 ± 0.416	–	0.58 ± 0.070	3.78 ± 0.072	
Ornithine (Orn)	5.82 ± 1.664	0.86 ± 0.070	0.14 ± 0.017	0.69 ± 0.011	0.65 ± 0.050
1 Methyl histidine (1 Mehis)	0.99 ± 0.014	1.98 ± 0.021	0.87 ± 0.037	1.40 ± 0.448	1.09 ± 0.011
3 Methyl histidine (3 Mehis)	11.97 ± 1.101	–	1.17 ± 0.045	–	–
Carnosine (Car)		–	1.06 ± 0.019	–	–
Hydroxyproline (Hypro)	4.60 ± 0.183	1.29 ± 0.101	1.33 ± 0.157	1.48 ± 0.021	1.17 ± 0.044
Citruline (Cit)	2.03 ± 0.532	–	1.26 ± 0.024	–	–

1 F, Fishmeal; TC, *T. catappa*; LM, *L. minor*; SM, *S. molesta*; TCLMSM, *T. catappa* + *L. minor* + *S. molesta*.

**Table 3:** Composition of diets and their proximate analysis. Data are given as Means ± SE (n = 3).

Ingredients (g/kg diet)	Diets					
	F^1^		FTC^1^	FLM^1^	FSM^1^	FTCLMSM^1^
Fishmeal	316.4		198.6	221.2	245.7	219.5
*Terminalia catappa* oil-cake	-----		198.6	-----	-----	73.2
*Lemna minor*	-----		-----	221.2	-----	73.2
*Salvinia molesta*	-----		-----	-----	245.7	73.2
Wheat flour	649.6		568.8	523.6	474.6	527.0
Cod liver oil	30.0		30.0	30.0	30.0	30.0
Vitamin-mineral premix^2^	4.0		4.0	4.0	4.0	4.0
Proximate analysis (g/kg)						
Dry matter^3^	928.6 ± 2.27	924.1 ± 2.15		939.2 ± 2.32	944.0 ± 1.17	925.3 ± 2.54
Crude protein	315.8 ± 1.50	313.0 ± 2.12		304.6 ± 1.55	303.5 ± 2.35	314.6 ± 1.25
Crude lipid	86.4 ± 2.55	87.3 ± 1.87		76.3 ± 1.24	79.8 ± 1.45	80.6 ± 1.25
Total carbohydrate^4^	454.5 ± 1.21	464.6 ± 2.25		468.9 ± 2.17	461.7 ± 1.2	453.5 ± 2.78
Crude ash	72.0 ± 2.12	59.2 ± 1.22		89.6 ± 1.17	99.2 ± 1.25	76.6 ± 2.15
Energy value (kcal/ kg)^5^	3858 ± 34.0	3896 ± 34.31		3780 ± 26.0	3778 ± 27.2	3798 ± 27.4

1 F, Fishmeal; FTC, Fishmeal + *T. catappa*; FLM, Fishmeal *+* L. *minor*; FSM, Fishmeal *+ S. molesta*; FTCLMSM, Fishmeal + *T. catappa* + *L. minor + S. molesta*.

2 Supradyan multivitamin tablets with minerals and trace elements contains (as mg/kg in diets): = Vitamin A (as acetate) 12; Cholecalciferol 0.1; Thiamine mononitrate, 40; Riboflavine 40; Pyridoxine hydrochloride, 12; Cyanocobalamin, 0.06; Nicotinamide, 400; Calcium pantothenate 65.20; Ascorbic acid 600; α-Tocopheryl acetate,100; Biotin, 1.00. Minerals: Tribasic calcium phosphate, 516; Magnesium oxide, 240; Dried ferrous sulphate, 128.16; Manganse sulphate monohydrate 8.12; Total phosphorus, 103.20. Trace elements: Copper sulphate pentahydrate 13.56; Zinc sulphate, 8.80; Sodium molybdate dihydrate, 1.00; Sodium borate 3.52.

3 Dry matter = Weight in g (1000 - Moisture) in 1 kg of feed.

4 Carbohydrate = 1000 - ([Moisture + Protein + Lipid + Ash] contents of 1 kg feed).

5 Energy (kcal/kg) = [(Crude protein g/kg × 4) + (Crude lipid g/kg × 9) + (Total Carbohydrate g/kg × 4)].

The amino acids contents of ingredients were determined with Automatic Amino Acid Analyzer l-8900 (Hitachi Co. Ltd., Tokyo, Japan). Briefly, the sample was digested with 6 N HCl at 110 °C for 22 h except methionine, cysteine and tryptophan. Digested sample was dried in Nitrogen Concentrator (PCi Analytic Private limited, Maharashtra, India). Then 0.02 N HCl was added in the dried sample and made the concentration of protein 0.5 mg/mL. The sample (1.5 mL) was taken in glass vial and kept in the Auto sampler. In determination column, a 20 μL sample was injected with a flow rate of 0.35 mg/mL and the column temperature was 30−70 °C. In reaction column, reaction temperature was 135 °C with a ninhydrin flow rate of 0.35 mg/mL. The ninhydrin derivatives of proline and hydroxyproline were monitored at 440 nm, while other amino acids were monitored at 570 nm. The amino acids were compared with standards and quantified (Wako Pure Chemical Industries Limited, USA). All samples of ingredients and diets were analyzed in triplicates.

### 2.2 Formulation of diets and culture of fish

Five different diets were prepared. The control diet was prepared with only fishmeal (F). Four plant-based diets were formulated with the proportion of fishmeal and plant ingredient maintained at 1:1. In FTC diet, fishmeal was blend with TC; in FLM, fishmeal was blend with LM; in FSM, fishmeal was blend with SM and diet FTCLMSM was a blend of fishmeal and all three plant ingredients *viz*. TC, LM and SM (Table 3). All dried ingredients were collected in appropriate amount and mixed properly before addition of oil; then sinking pelleted diets (1 mm die) were prepared with a Twin-Screw-Extruder (Basic Technology, Kolkata, India). Diets were specially formulated to a fixed dietary protein content of 300 g/kg with equal amounts of plant material and fishmeal. Thus, the plant ingredients replaced 370, 300, 220 and 310 g/kg of fishmeal in FTC, FLM, FSM and FTCLMSM diets, respectively compared to the F diet.

Indian major carp rohu *Labeo rohita* were obtained from Chatterjee Brothers’ Fish Farm, West Bengal. Fish (initial average weight: 10.66 ± 0.53 g) were randomly distributed in 15 glass aquaria (10 fish/50 L aquarium) in triplicate in laboratory facility of University of Delhi. Each aquarium was connected with an external, mechanical filter (Sera fil bioactive 130, Germany). Water from each fish culture unit came to the mechanical filter and after filtration, the water was back to the culture unit. Rohu were acclimated at 25 °C for 7 days to mitigate handling stress. Rohu were maintained on a 12 h light: 12 h dark regime throughout the study period. Fish were cultured under five different feeding regimes: F, FTP, FLM, FSM and FTCFLMFSM and feed was given at a rate of 3% of body weight every day. The amount of feed was adjusted as the weight of fish increased during the study period. The total amount of feed was divided in two parts and delivered at 9.00 a.m. and 5.00 p.m. Excess food was collected after 1 h of each feeding and it was used for the determination of actual feed consumption rate. All fish were harvested after 90 days of culture. Survival rate and final body weight of fish were recorded. The study was conducted following the guidelines of Animal Ethics Committee (IAEC), Department of Zoology, University of Delhi, Delhi, India (DU/ZOOL/IAEC-R/2015/07).

### 2.3 Water quality

Water samples were collected at weekly interval (4 samples/ month) from each treatment (3 replicates/treatment) and twelve samples were collected during 90 days culture period. There were 36 samples/ treatment (3 replicates x 12 samples). Water quality parameters including temperature, pH, conductivity and dissolved oxygen levels of aquaria were monitored regularly using a probe connected to a portable meter (IntelliCAL LDO101, Hach, USA). Similarly, ammonia was monitored using appropriate probe (HQ40d Multiparameter, Hach, USA). Nitrite (4500-NO_2_^−^) and nitrate (4500-NO_3_^−^) were measured following the methods of American Public Health Association (APHA) (2012).

### 2.4 Sampling of fish

After 90 days of feeding trial, fish were fasted for 24 h. All fish were weighed and then anaesthetized with tricaine methanesulphonate MS-222; Sigma, USA. Fish were dissected on a glass plate maintained at 0 °C. The digestive tract of individual fish two fish per replicate; 2 × 3 replicates = 6 fish per treatment was collected, rinsed with chilled distilled water, blot dried and weighed. Then the entire digestive tract was homogenized in chilled distilled water 1:10 to maintain neutral pH of extract as this extract was used for various enzyme assays at different pHs. The homogenate was centrifuged at 10,000 x g for 15 min at 4 °C Sigma 3K30, Germany and the supernatant collected and used for enzyme activity study. Total soluble protein was measured following the method of Bradford (1976) using bovine serum albumin (Sigma, St Louis, USA) as a standard (1 mg/mL).

All enzymes were assayed using fluorometric methods (Fluoremeter, BioTek Synergy H1, USA). Amylase activity was measured with EnzChek@ Ultra Amylase Assay kit (E33651, Invitrogen, USA) with fluorescence measured at 485 nm for excitation and 520 nm for emission. Total protease activity was measured using EnzChek@ Protease Assay kit (E6638, Invitrogen, USA) with fluorescence measured at 485 nm (excitation) and 530 nm (emission). Trypsin activity was estimated using Na-benzoyl-l-arginin-methyl-coumarinylamide (Sigma, USA) as substrate (Ueberschär, 1988) with fluorescence measured at 380 nm (excitation) and 440 nm (emission). Chymotrypsin activity was measured following the method of Cao et al. (2000) using succinyl-Leu-Val-Tyr-4-methyl-coumaryl-7-aminde (Sigma, USA) as substrate and fluorescence measured at 380 nm (excitation) and 450 nm (emission). Neutral lipase activity was measured using 4-methylumbelliferyl butyrate (4-MUB, Sigma, USA) as substrate (Roberts, 1985) with fluorescence recorded at 365 nm for excitation and 450 nm for emission.

### 2.5 Specific growth rate, weight gain, feed intake and feed conversion ratio

The specific growth rate (SGR), weight gain (WG), feed intake (FI) and feed conversion ratio (FCR) were calculated as follows:

SGR (%) = (In Final body weight - In Initial body weight) × 100/ Duration of expriment.

WG (%) = 100 [(Final body weight - Initial body weight)/ Initial body weight].

FI = 100 x Total feed fed (dry matter)/ [(Initial weight *+* Final weight + Dead fish weight)/ 2 x days].

FCR = Dry weight of feed consumed by individual fish during experiment/ Wet weight gain of individual fish

### 2.6 Statistical analysis

Chemical composition of feed ingredients, diets and water quality parameters were given as Means ± SE of three replicates and analyzed using one-way analysis of variance (ANOVA). Amino acids composition of ingredients and proximate composition of feeds were given as Means ± SE of three replicates. Performance parameters (IBW, FBW, WG, SGR, FI and FCR) and digestive enzyme activities (amylase, protease, trypsin, chymotrypsin and lipase) were given as means with pooled standard error (pSEM), using the aquarium as the experimental unit, and analyzed using one-way ANOVA and Duncan's multiple range test (Montgomery, 1984). Statistical analyses were performed using the Statistics 22 program (SPSS, 2013). Statistical significance was accepted at P < 0.05 level.

## 3 Results

### 3.1 Composition of ingredients

Analyses of chemical composition of raw ingredients showed that there was variation in the composition (Table 1). Protein, lipid and ash contents were significantly higher in fishmeal, almond oil-cake and duckweeds, respectively compared to the other ingredients. The amino acid profiles showed that all essential and non-essential amino acids were present in almond oil-cake, duckweeds, water fern and fishmeal although there was variation in their amount in different ingredients (Table 2). The highest amount of essential amino acids was found in fishmeal followed by duckweed, other than histidine content, that was higher in almond oil-cake compared to duckweed. Similar to the essential amino acids, non-essential amino acids contents were highest in fishmeal compared to other ingredients, other than glutamic acid that was highest in almond oil-cake. Some free amino acids such as sarcosine, α-amino-n-butaric acid, 3-methyl histidine and citruline were absent in almond oil-cake and water fern, but were present in fishmeal and duckweed.

### 3.2 Water quality

There were no significant differences in temperature, pH, dissolved oxygen, ammonia, nitrite, nitrate and conductivity of water in five different treatments throughout the study period (Table 4). Water temperature and pH ranged from 25.0 ± 0.5–27.0 ± 1.0 °C and 7.85–8.48 in different treatments, respectively during the study period. Dissolved oxygen level was always above 5 mg/L regardless of feeding regimes. Ammonia, nitrite and nitrate levels ranged from 0.54 - 0.69, 0.21 - 0.25 and 2.28–2.32 mg/L, respectively in different treatments. Ammonia and nitrite levels were below 1.0 mg/L in all treatments throughout the study period. Conductivity ranged from 609.41 to 632.00 μS/cm in various treatments.

**Table 4:** Dissolved oxygen, ammonia, nitrite, nitrate and conductivity of water found in different treatments during 90 days of culture. Water quality was monitored at weekly interval in various treatments (4 samples/month/treatment; 12 samples/ treatment in 3 months; 3 replicates/treatment). Data are provided as Means ± SE (n = 3).

Parameters	F^1^	FTC^1^	FLM^1^	FSM^1^	FTCLMSM^1^
Dissolved oxygen (mg/L)	6.98 ± 0.103	6.98 ± 0.184	6.79 ± 0.053	7.12 ± 0.069	7.10 ± 0.025
Ammonia (mg/L)	0.67 ± 0.009	0.54 ± 0.028	0.68 ± 0.057	0.57 ± 0.009	0.69 ± 0.025
Nitrite (mg/L)	0.23 ± 0.005	0.22 ± 0.005	0.21 ± 0.002	0.23 ± 0.002	0.25 ± 0.002
Nitrate (mg/L)	2.28 ± 0.039	2.29 ± 0.029	2.32 ± 0.024	2.30 ± 0.028	2.31 ± 0.026
Conductivity (μS/cm)	613.91 ± 4.763	610.08 ± 2.068	632.00 ± 3.079	609.41 ± 1.683	611.16 ± 4.041

1 F, Fishmeal; FTC, Fishmeal + *T. catappa*; FLM, Fishmeal *+ L. minor*; FSM, Fishmeal *+ S. molesta*; FTCLMSM, Fishmeal + *T. catappa* + *L. minor + S. molesta*.

### 3.3 Survival and growth of fish

There was hundred percent survival of rohu cultured under five different feeding regimes. All fish survived. There was no significant difference in the body weight of fish at the beginning of the study. The final body weight was significantly higher in rohu fed diet FLM compared to the fish fed the other diets (Table 5). There were no significant difference between the final body weights of fish fed diets FTC and FTCLMSM. Final body weight was lowest in fish fed diet FSM. Consequently, the weight gain and specific growth rate of rohu showed the similar trend. Highest SGR was found in fish fed diet FLM compared to the fish fed the other diets. Feed intake and feed conversion ratio showed the opposite trend. FCR was significantly lower in rohu fed diets FLM compared to fish fed the other diets.

**Table 5:** Growth performance and feed conversion ratio of *L. rohita* fingerlings fed with five different diets for 90 days. There were three replicates/treatment and 10 fish/replicate (10 × 3 = 30 fish/treatment). Means with different superscripts in the same row are significantly different.

Parameters	Diets					pSEM	P-value
	F^1^	FTC^1^	FLM^1^	FSM^1^	FTCLMSM^1^		
IBW (g)^1^	10.66 ^a^	10.66 ^a^	10.66 ^a^	10.66 ^a^	10.66 ^a^	0.007	0.998
FBW (g)^1^	20.89^c^	21.47^b^	22.45^a^	20.48^c^	21.37^b^	0.132	<0.001
WG (%)^2^	96.00^c^	101.41^b^	110.60^a^	92.12^c^	100.50^b^	0.181	<0.001
SGR (%)^3^	0.75^c^	0.78^b^	0.83^a^	0.73^c^	0.77^b^	0.062	<0.001
FI (g/100 g BW/day)^4^	1.69^b^	1.64^c^	1.61^d^	1.71^a^	1.64^c^	0.001	<0.001
FCR^5^	2.34^b^	2.22^c^	2.03^d^	2.44^a^	2.24^c^	0.003	<0.001

1 F, Fishmeal; FTC, Fishmeal + *T. catappa*; FLM, Fishmeal *+ L. minor*; FSM, Fishmeal *+ S. molesta*; FTCLMSM, Fishmeal + *T. catappa* + *L. minor + S. molesta.* IBW = Initial body weight, FBW = Final body weight.

2 WG = Weight gain (%) = 100 [(Final body weight - Initial body weight)/ Initial body weight].

3 FI = Feed intake = 100 x Total feed fed (dry matter)/ [(Initial weight *+* Final weight + Dead fish weight)/ 2 x days].

4 SGR = Specific growth rate (%) = (In Final body weight - In Initial body weight) × 100/ Duration of expriment.

5 FCR = Food conversion ratio = Dry weight of feed consumed by individual fish during experiment/Wet weight gain of individual fish.

### 3.4 Enzyme activities

Amylase activity was significantly higher in rohu fed diet FLM compared to fish fed the other diets (Table 6). This group was followed by fish fed diets FSM, FTCLMSM and FTC with lowest amylase activity in fish fed diet F. Total protease activity was significantly higher in rohu fed diets FTC and F compared to fish fed the other diets. There was no significant difference in total protease activity between these two former treatments. Significantly higher trypsin activity was recorded in rohu fed the duckweed-based diet compared to fish fed other diets. A similar trend was found with chymotrypsin activity with highest activity observed in fish fed diet FLM. Lowest trypsin and chymotrypsin activities were recorded in rohu fed diet FTC. In contrast, it was interesting that lipase activity was significantly higher in rohu fed almond oil-cake-based diet compared to fish fed other diets. This group was followed by rohu fed diet F, the fishmeal-based diet.

**Table 6:** Amylase, protease, trypsin, chymotrypsin and lipase activities found in *L. rohita* cultured in five different feeding regimes. There were three replicates/treatment and two fish/replicate (2 × 3 = 6 fish/treatment). Means with different superscripts in the same row are significantly different.

Parameters	Diets					pSEM	P-value
	F^1^	FTC^1^	FLM^1^	FSM^1^	FTCLMSM^1^		
Amylase (mU/mg protein/min)	23.30^d^	41.30^c^	64.92^a^	48.77^b^	44.70^bc^	0.757	<0.001
Protease (Fluorescence change/unit)	57.87^a^	58.19^a^	53.95^c^	53.25^c^	55.20^b^	0.515	0.015
Trypsin (μM AMC/mg protein/min)	32.20^d^	23.00^e^	76.63^a^	55.59^b^	42.90^c^	0.770	<0.001
Chymotrypsin (μM AMC/mg protein/min)	21.20^c^	15.10^d^	29.29^a^	15.46^d^	24.70^b^	0.360	<0.001
Lipase (μM 4-MU/mg protein/min)	12.37^b^	19.09^a^	9.31^c^	7.90^d^	10.26^c^	0.277	<0.001

1 F, Fishmeal; FTC, Fishmeal + *T. catappa*; FLM, Fishmeal *+ L. minor*; FSM, Fishmeal *+ S. molesta*; FTCLMSM, Fishmeal + *T. catappa* + *L. minor + S. molesta*.

## 4 Discussion

In the present study, highest growth was found in *L. minor* supplemented diet fed rohu. Earlier study showed that the supplementation of 25 % *A. microphylla* and *A. pinnata* mixture in diet enhanced the growth and SGR of rohu (Datta, 2011). Feeding of raw *Wolffia globosa*, the smallest duckweed to rohu fry showed better growth compared to the fish fed with formulated diet (Pradhan et al., 2019). Whereas, Stadtlander et al. (2019) reported that incorporation of another duckweed *Spirodela polyrhiza* at two levels of 6.25 and 12.5 % in the feed of rainbow trout affected the growth after 4 weeks of feeding. In the present study, supplementation of fish meal along with duckweed met the nutritional requirements of rohu. A lower FCR value showed that diet FLM was also utilized more efficiently in rohu compared to the other diets.

The study of chemical and amino acid compositions of almond oil-cake, duckweed and water fern largely showed the nutritional values of these ingredients as fish feed. The present study confirmed the earlier findings (Ahrens et al., 2005; Sharma et al., 2016; Chakrabarti et al., 2018). The presence of essential, non-essential and free amino acids in duckweed might influence the growth of rohu, despite the fact that their amounts were less in duckweed compared to fishmeal. Certainly, based on published amino acid requirements for rohu, duckweed protein could satisfy almost all the requirements. The essential amino acids requirements of rohu are reported as follows: arginine 2.30, histidine 0.90, isoleucine 1.20, leucine 1.50, lysine 2.27, methionine 1.42, phenylalanine 1.48, threonine 1.71, tryptophan 0.45 and valine 1.50 % of diet (Food and Agriculture Organization (FAO), 2013).

Amylase, trypsin and chymotrypsin activities were significantly higher in rohu fed diet FLM compared to fish fed other diets. The efficient enzyme activities in FLM might result in better FCR compared to the other feeding regimes in the present study. Earlier study showed that supplementation of 25 % *I. aquatica* leaf meal (fermented with bacteria) enhanced the α-amylase activity in rohu (Ali and Kaviraj, 2018). An *in vitro* digestibility study of almond oil-cake, duckweed and water fern showed the high degree of hydrolysis (DH%) of these raw ingredients with the digestive juices of rohu and common carp (Sharma et al., 2016). The effect of diet composition on digestive enzyme activities was found in the present study. Among the different ingredients used for diet formulation, the highest amount of lipid was found in the almond oil-cake and highest lipase activity was found in rohu fed with diet FTC, followed by fish fed with the diet F. In catla *Catla catla* larvae, effect of different type of diets was recorded (Meetei et al., 2014). Baragi et al. (2002) found that incorporation of raw and fermented (with *Bacillus* sp.) leaf meal of *L. polyrhiza* resulted in replacement of 10 and 30 % fishmeal, respectively in diet of rohu fingerlings. In the present study, in diet FLM, 300 g/kg (30 %) of fishmeal was replaced with raw duckweed compared to the fishmeal-based control diet. Application of extrusion technique for the preparation fish feed increased digestibility and nutrient utilization of the ingredients (Stadtlander et al., 2019).This resulted in better performances of rohu fed with diet supplemented with raw duckweed.

## 5 Conclusions

The present study demonstrated that duckweed *Lemna minor* is a nutrient rich and digestible feed ingredient for carp rohu. The prepared pelleted feed may replace fishmeal up to 300 g/kg of feed and, thereby, reduce the cost.

## Author contributions

RC, DRT, JGS: designed the study; RKG, AKS, RC, JGS: cultured the plants, prepared diets and conducted experiment with fish; analyzed samples; RC, DRT, JGS: wrote the manuscript; RKG, AKS: prepared the tables.

## Declaration of Competing Interest

The authors declare that there is no conflict of interest.
